# Droplet digital PCR shows the D-Loop to be an error prone locus for mitochondrial DNA copy number determination

**DOI:** 10.1038/s41598-018-29621-1

**Published:** 2018-07-30

**Authors:** Brian Li, Sonal Kaushik, Pola Kalinowski, BaRun Kim, Cynthia Gershome, Joyce Ching, Damon Poburko

**Affiliations:** 1 Department of Biomedical Physiology and Kinesiology, Simon Fraser University, Burnaby, Canada; 2 Centre for Cell Biology, Development, and Disease, Simon Fraser University, Burnaby, Canada

## Abstract

Absolute quantification of mitochondrial DNA copy number (mCN) provides important insights in many fields of research including cancer, cardiovascular and reproductive health. Droplet digital PCR (ddPCR) natively reports absolute copy number, and we have developed a single-dye, multiplex assay to measure rat mCN that is accurate, precise and affordable. We demonstrate simple methods to optimize this assay and to determine nuclear reference pseudogene copy number to extend the range of mCN that can be measured with this assay. We evaluated two commonly used mitochondrial DNA reference loci to determine mCN, the ND1 gene and the D-Loop. Harnessing the absolute measures of ddPCR, we found that the D-Loop amplifies with a copy number of ~1.0–1.5 relative to other sites on the mitochondrial genome. This anomalous copy number varied significantly between rats and tissues (aorta, brain, heart, liver, soleus muscle). We advocate for avoiding the D-Loop as a mitochondrial reference in future studies of mCN. Further, we report a novel approach to quantifying immunolabelled mitochondrial DNA that provides single-cell estimates of mCN that closely agree with the population analyses by ddPCR. The combination of these assays represents a cost-effective and powerful suite of tools to study mCN.

## Introduction

Eukaryotic mitochondria contain a circular genome of ~16 kb encoding two rRNAs, 22 tRNAs, 13 genes for electron transport chain subunits and at least two hormone-like peptides^1,2^. Changes in mitochondrial DNA (mtDNA) copy number (mCN) and deletion frequency are linked to the natural aging process^3^, elevated blood pressure^4,5^, and diseases including cancer^6^ and Alzheimer’s disease^7^. Until recently, the most precise method to quantify mCN was quantitative or real-time polymerase chain reaction (qPCR)^8^. Using hydrolysis probes, multiplex qPCR simultaneously measures a mtDNA locus relative to a nuclear reference gene. While qPCR can be performed using reference standards to determine absolute copy number, these standards can introduce copy number errors particularly when using circular plasmid DNA^9^. Thus, there exists a need for assays to report absolute mCN in a manner that is clearly standardized within the research community.

Droplet digital PCR (ddPCR) is a technique in which the polymerase chain reaction is performed on a sample that has been encapsulated into oil droplets, such that the probability of a droplet containing zero or a small number of template molecules follows a Poisson distribution^10^. As with qPCR, template amplification is detected with a DNA-binding dye or hydrolysis probes. Unlike qPCR, ddPCR calculates that absolute concentration of template by using Poisson statistics to determine the quantal content of template in individual dropletized reactions. As a consequence, reference standards and serial reaction dilutions are not required, which reduces operational costs^11^ and eliminates potential errors introduced in the generation of reference standards. ddPCR also offers improved precision^12^ and sensitivity^10^ over qPCR. An additional benefit of ddPCR is the ability to perform multiplex reactions using a single DNA binding dye (e.g. EvaGreen)^13,14^, providing further cost savings over probed-based multiplex reactions^11^. While ddPCR is able to detect exceedingly rare mutations (1-in-10^5^)^10^, duplex ddPCR assays have a limited effective range limiting their precision when measuring mCN in high copy number tissues (i.e. > ~3000 copies/cell)^15^. In spite of this limitation, the benefits of ddPCR provide significant improvements over qPCR for measurements of mCN^10,15,16^.

**Figure 1:**
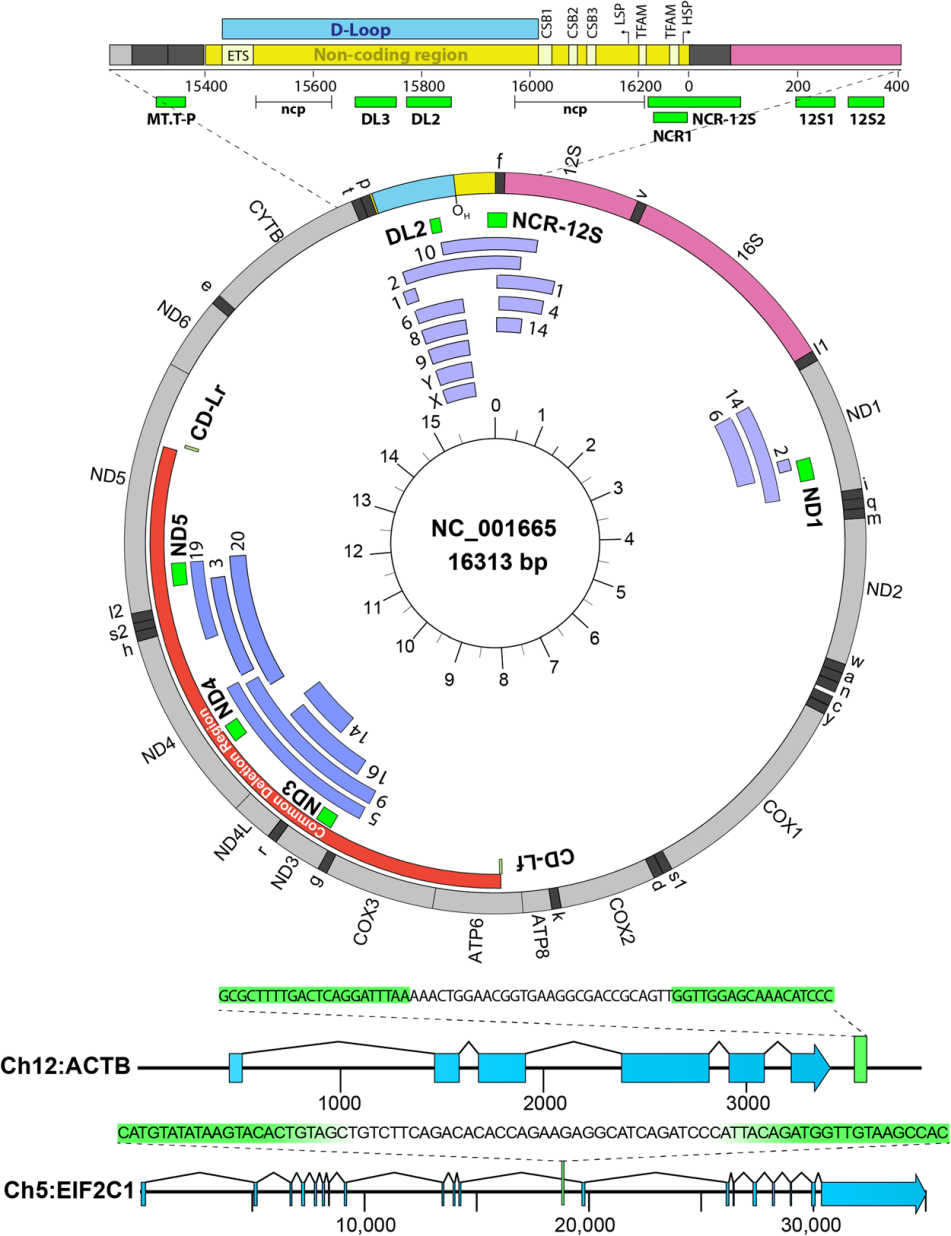
mtDNA and location of primers and major elements. Labelling on outer ring represents mtDNA genes based on accession number NC_001665 (16,313 bp). Innermost ring denotes thousands of base pairs clockwise. Red inner ring shows the common deletion region. Green bands show amplicons of primers used in our studies. Additional arcs are numbered for chromosomes with NUMTS at the aligned position of mtDNA. The non-coding region is illustrated with the location of the D-Loop between the early termination sequence (ETS) and Conserved Sequence Blocks (CSB1–3) and transcript initiation sites of the light (LSP) and heavy (HSP) strands and their predicted TFAM binding sites^49^.

We set out (1) to optimize a single-dye, multiplex assay to measure absolute mCN, (2) to determine the limit of detection for this assay and (3) to determine an optimal mtDNA locus for mCN determination. Our literature analysis showed that numerous mtDNA loci are used to assess mCN; the most common being the D-Loop within the mitochondrial DNA control region (see Fig. 1) and the ND1 gene followed by tLeucine and the cytochrome B (CytB) gene. We did not encounter a systematic analysis of the optimal loci for mCN determination. Thus, we tested whether ND1 and the D-Loop provide equivalent references for analyzing mCN or deletion frequency. Further, we assessed whether quantification of mCN by imaging immunolabelled mtDNA nucleoids could provide an estimate of mCN that is consistent with mCN measured by ddPCR. Having validated the EvaGreen assay, we sought to compare mCN in cultured vascular smooth muscles versus intact blood vessel as a measure of the mitochondrial biogenesis that occurs when smooth muscle cells are cultured and in pathologic conditions like atherosclerosis and hypertension^17^.

Harnessing the absolute quantification of ddPCR, we show that primers targeting the D-Loop report anomalous and experimentally labile mCN relative to other mtDNA loci. The EvaGreen mCN assay provides precision on par with hydrolysis probe duplex assays, while using a nuclear reference of non-unitary copy number can extend the dynamic range of mCN detection. In parallel, we describe imaging methods to estimate single-cell mCN that correlates closely with ddPCR results. Using these methods, we report that aorta contains significantly lower mCN than a variety of other tissues and that mCN is approximately three-fold higher in cultured aorta smooth muscle cells than in freshly isolated tissue. We believe that the methods reported below will be of significant value to others studying mCN.

## Methods

### Animal Tissue and Cell Culture

All experimental procedures conformed to the Canadian Council on Animal Care (CCAC) Guide to the Care and Use of Experimental Animals and were approved by the Simon Fraser University Animal Care Committee (UACC). Sprague Dawley rats (male and female, 2–24 months of age, Envigo) were euthanized by asphyxiation with CO_2_ (20–30%). Tissues were immediately removed and placed in ice cold HEPES (cold 4-(2-hydroxyethyl)-1 piperazineethanesulfonic acid) dissection buffer (in mM: 140 NaCl, 5 KCl, 5 HEPES, 0 CaCl_2_, 1 MgCl_2_, 10 glucose, pH 7.4) during collection. Tissues were then patted dry, chopped into small pieces and frozen at −80 °C. A7r5 rat aorta smooth muscle cells were purchased from the American Type Culture Collection (ATCC, CRL-1444). Cells were grown in cell culture treated culture flasks in DMEM (Life Technologies,10569044) containing 25 mM glucose and supplemented with 10% fetal calf serum (Life Technologies) and antibiotics (Life Technologies, 15140–122) at 37 °C in a 5% CO_2_ incubator. For genomic DNA isolation (gDNA), cells were sub-cultured on 6-well plates. For imaging, cells were grown in 24-well plates on 12 mm #0 glass coverslips coated with laminin (2 µg / coverslip).

### Immunofluorescence & Imaging

Cells were labelled with 50 nM MitoTracker Orange CMTMRos (Life Technologies, M-7510) for 45 minutes at 37 °C in culture media. Cells were then fixed in 4% paraformaldehyde (Electron Microscope Science), and permeabilized for 10 minutes with 0.1% Triton-X100. Cells were blocked with 4% normal goat serum before being labelled with 0.17 µg/ml anti-DNA antibody (EMD Millipore, CBL186) overnight at 4 °C. Fixative and all subsequent solution were based on phosphate buffered solution. Excess primary antibody was removed with three PBS washes before staining with Goat anti-IgM Alexa-488 (2 µg/ml¸ Life Technologies, A21042). Nuclei were labelled with Hoechst-33342 (1 µg/ml) for 20 minutes before mounting in ProLong Gold (Life Technologies). Cells were imaged on a Nikon TiE inverted microscope with a 100x CFI Plan Apo Lambda 1.45 NA objective using a Zyla 5.5 CMOS camera (Andor). Images were analyzed using ImageJ^18^.

### Animal Tissue Collection and Genomic DNA Isolation

To facilitate DNA extraction, tissue samples were pulverized using a custom made stainless steel mortar with a round bottom cup and a stainless-steel pestle with a slightly smaller radius of curvature. The mortar was cooled in liquid nitrogen and the pestle was kept on dry ice to prevent thawing of tissue during pulverizing. Approximately 100 mg of tissue at a time was pulverized into a fine powder using a hammer and the pestle. Genomic DNA isolation of A7r5 cells and rat tissue (20–40 mg) were performed using the GeneJET Genomic DNA Purification Kit (Thermo Scientific) as per the product manual. The concentration of the collected DNA was measured in quadruplicate on a Nanodrop 1000 spectrophotometer. Outlier values were discarded before calculating mean DNA concentration.

### Primer Design

Primers were purchased from Integrated DNA Technologies. Primer sequences are previously described for the non-coding region (NCR-12S), beta actin (BA), and common deletion flanking (CD-L) primers^19^. Binding locations of the primers and the amplicon sequences were verified using Primer-BLAST. The EIF2C1-HEX probe was purchased from Bio-Rad (cat. # 10031229). Figure 1 shows the loci of the BA and EIF2C1 amplicons relative to each nuclear and mitochondrial gene. Additional primers for the D-Loop (DL2), the ND1 gene, and three sites within the 4997-bp mitochondrial common deletion (ND3, ND4 and ND5), were designed using NCBI’s Primer BLAST. Amplicon lengths were limited to 70–200 bp as per Bio-Rad’s ddPCR guidelines with an optimal annealing temperature of 60 °C. In the case of ND1 and ND5, several primers were screened, and the primer pairs producing the least spreading in droplet intensity and a clear single band of positive droplets were selected. Primers were designed against the rat mitochondrial genome JX105355, and cross-referenced for compatibility with NC_001665 and X14848. Primer sequences are shown in Table 1.

**Table 1:** Primer sequences.

Gene / target	5′-Forward-3′	Amplicon Length
	5′-Reverse-3′	
12S1	GCTCAAGACGCCTTGCCTA	78
	AGTCAAACTTTCGTTCATTGCT	
12S2	ATTTCGTGCCAGCCACCG	71
	TAGTTGGCACGTTTTACGCC	
BA	GGGATGTTTGCTCCAACCAA	69
	GCGCTTTTGACTCAGGATTTAA	
DL2	GGTTCTTACTTCAGGGCCATCA	83
	GATTAGACCCGTTACCATCGAGAT	
DL3	TCCGTGAAATCAACAACCCG	77
	CAGTATAGTCACCCCCAGGA	
MT.T-P	AAGAGTCAATCTTCTCAGGACA	55
	TTGATGGTGGGGAGGTAGTT	
ND1	TTAACGTCGAATACGCCGCA	144
	AGCTGGTTGAGTATAATTCAGGGT	
ND3	ACTCCGAAAAAGCAAACCCAT	137
	GAGGGGGAGTAGTAAGGCGAT	
ND4	GGCAACCAAACAGAACGCTT	145
	GGTGTGTTGTGAGGGAGAGG	
ND5	TCCTATCAGTAGCCCTATTCGT	82
	CGGTTAATGTGGGGGTCAGA	
NCR1	TCCCGACACAAAATCTTTCCT	63
	GGAATTTTCTGAGGGTAGGCA	
NCR-12S	GGTTCTTACTTCAGGGCCATCA	175
	GATTAGACCCGTTACCATCGAGAT	

### End-point PCR

End-point PCR reactions were prepared with 1.0 µl of genomic DNA (20 ng) from the sample of interest, 4 µl of Phusion HF buffer, 2.4 µl of 10 mM dNTPs (Thermo), 2 µl of 5 µM forward and reverse CD-L primers, 0.2 µl of Phusion DNA polymerase (Thermo), and 8.4 µl of autoclaved, double distilled water. Reactions were cycled as follows: Initial denaturation (98 °C, 30 s), 30 cycles of denaturation (98 °C, 10 s), annealing (66.5 °C, 30 s), extension (72 °C, 169 s), then a final extension (72 °C, 10 min) and hold at 4 °C. Reaction products (5 µl of reaction plus 1 µl of loading dye) and O’GeneRuler 1 kb DNA ladder (5 µl, Thermo, SM1163) were loaded into 5 mm lanes on a 1% TAE agarose gel containing 20 ng/ml ethidium bromide and run for 50 minutes at 4.7 V/cm.

### Real-time PCR

qPCR was performed on a CFX96 Real-Time System + C1000 Touch Thermal Cycler (Bio-Rad). A7r5 gDNA was assayed at 10-fold dilutions in triplicate. 20 µl reactions contained 0.01–10 ng DNA, 100 nM CD-L primers^19^, 10 µl SYBR master mix (#4472942, Applied BioSystems) and balance distilled water. Reactions were cycled as: denaturation/HotStart (95 °C, 180 s), then 39 cycles of denaturation (95 °C, 15 s), anneal/read (58 °C, 40 s), and extension (72 °C, 40 s) followed by a 65–95 °C melt curve. Results were analyzed using CFX Maestro Software (Bio-Rad), and relative abundance was calculated by Levak’s method using Excel (Microsoft).

### Droplet Digital PCR

We performed ddPCR reactions as described in the Bio-Rad Droplet Digital PCR Applications Guide (www.bio-rad.com/webroot/web/pdf/lsr/literature/Bulletin_6407.pdf). Briefly, 25 µl reactions were prepared with 2.0 µl of the genomic DNA (0.25–10 ng), 2.0 µl primers or probes, 5 units of FastDigest™ HIND III restriction enzyme (Fermentas) per ≤ 1 ng of DNA, and 12.5 µl supermix (EvaGreen dye #1863010, Bio-Rad), with the balance being autoclaved, double distilled water. Assays for EIF2C1 also used EvaGreen supermix, and Bio-Rad EIF2C1 probe (cat. # 10031229, Bio-Rad). We vortexed and centrifuged the master mix before adding DNA or primer aliquots in strip tubes which were again vortex and centrifuged. We then reverse pipetted 20 µl per well into droplet generator cartridges (1864008, Bio-Rad) along with 70 µl of Droplet Generation Oil for EvaGreen. To improve assay reproducibility, particularly for singleplex reaction, we (i) diluted forward and reverse primers into a single mixture at 12.5x final concentration so that the 2 µl of primers added to reaction gave a consistent final concentration, and (ii) we measured gDNA concentrations four times to minimize the impact or read-to-read variability on the NanoDrop. We transferred 40 µl of dropletized reactions to a 96-well plate, heat sealed the plate (5–7 s, 180 °C, Bio-Rad PX1 PCR Plate sealer), and cycled with the following program: Enzyme activation (95 °C, 5 min), 40 cycles of denaturation (95 °C, 30 sec) and annealing-extension (56 °C, 90 sec), signal stabilization (4 °C, 5 min + 90 °C, 5 min), hold at 4 °C. For all steps, a ramp rate of 2 °C/sec was used. Dropletized PCR reactions were analyzed on the QX200^TM^ Droplet Reader.

### Statistical Analysis

Poisson-corrected determination of template concentrations were calculated in QuantaSoft (v1.7.4, Bio-Rad) after manual gating of positive and negative droplet populations. Template concentrations and ratio values were further analyzed in Excel 2016 (v1701, Microsoft, Redmond, WA, USA) and JMP® (v12, SAS Institute Inc., Cary, NC, 1989–2007). Figures were prepared in Prism v6.07 (GraphPad Software, La Jolla, CA, USA). T-tests were performed in Prism and JMP®, and ANOVAs were performed using JMP® and SPSS (v22.0, IBM Corp., Armonk, NY, USA). Curving fitting was performed in Prism (GraphPad) by least squares optimization.

### Data availability

Data and associated protocols will be made available upon request to the D. Poburko.

## Results

### Analysis of NUMTS

We first assessed whether nuclear mitochondrial DNA segments (NUMTS) were likely to be amplified by our primers. We performed BLAST searches flanking each amplicon against the *Rattus Norvegicus* Rnor6.0 genome. We identified numerous NUMTS not annotated in Rnor6.0 (Fig. 1). The region around DL primers had NUMTS on chromosomes 1, 2, 4, 6, 8, 9, 10, 14, X and Y. The DL2 amplicon resembled NUMTS on chromosomes 2, 6, 8, 9, Y and X. The NCR-12S amplicon was only spanned by NUMTS on chromosomes 2 and 10. The ND1 gene had NUMTS on chromosomes 2, 6 and 14, but only the chromosome (Chr) 14 NUMT spanned the ND1 amplicon. For the ND3, ND4 and ND5 primer binding sites, we found NUMTS on chromosomes 3, 5, 9, 14, 16, 19 and 20. The Chr 5 and 9 NUMTS spanned the ND3 and ND4 amplicons, and the Chr 19 and 20 NUMTS spanned the ND5 amplicon. Most primers had 1–5 mismatches with the corresponding NUMTS. The upper limit of potential mCN due to NUMTS per nuclear genome are: 2 NCR-12S, 6 DL2, 1 ND1, 2 ND3, 2 ND4, 2 ND5.

### Multiplex Assay Optimization

A common annealing and extension temperature provided specific amplification of each primer pair as indicated by the amplification of a single population of positive droplets with a Gaussian distribution of intensities and minimal droplets with intermediate intensity (“rain”). For the DL2, Eif2C1, BA and ND3 primer pairs, eight 20 µl reactions were prepared, each containing 10 µl of EvaGreen supermix, 1.0 µl of FastDigest HindIII (Thermo), 1.0 ng of A7r5 genomic DNA (1.0 ng/µl), 2.0 µl of 2.0 μM forward and reverse primer, and 6 µl of autoclaved water. Parallel reactions were run on a 55 to 62 °C gradient. Rain in reactions for EIF2C1, BA and ND3 increased at annealing temperatures above ~57 °C (Fig. S1), while 56 °C produced single band of positive droplets with minimal rain and was used for future assays.

**Figure 2:**
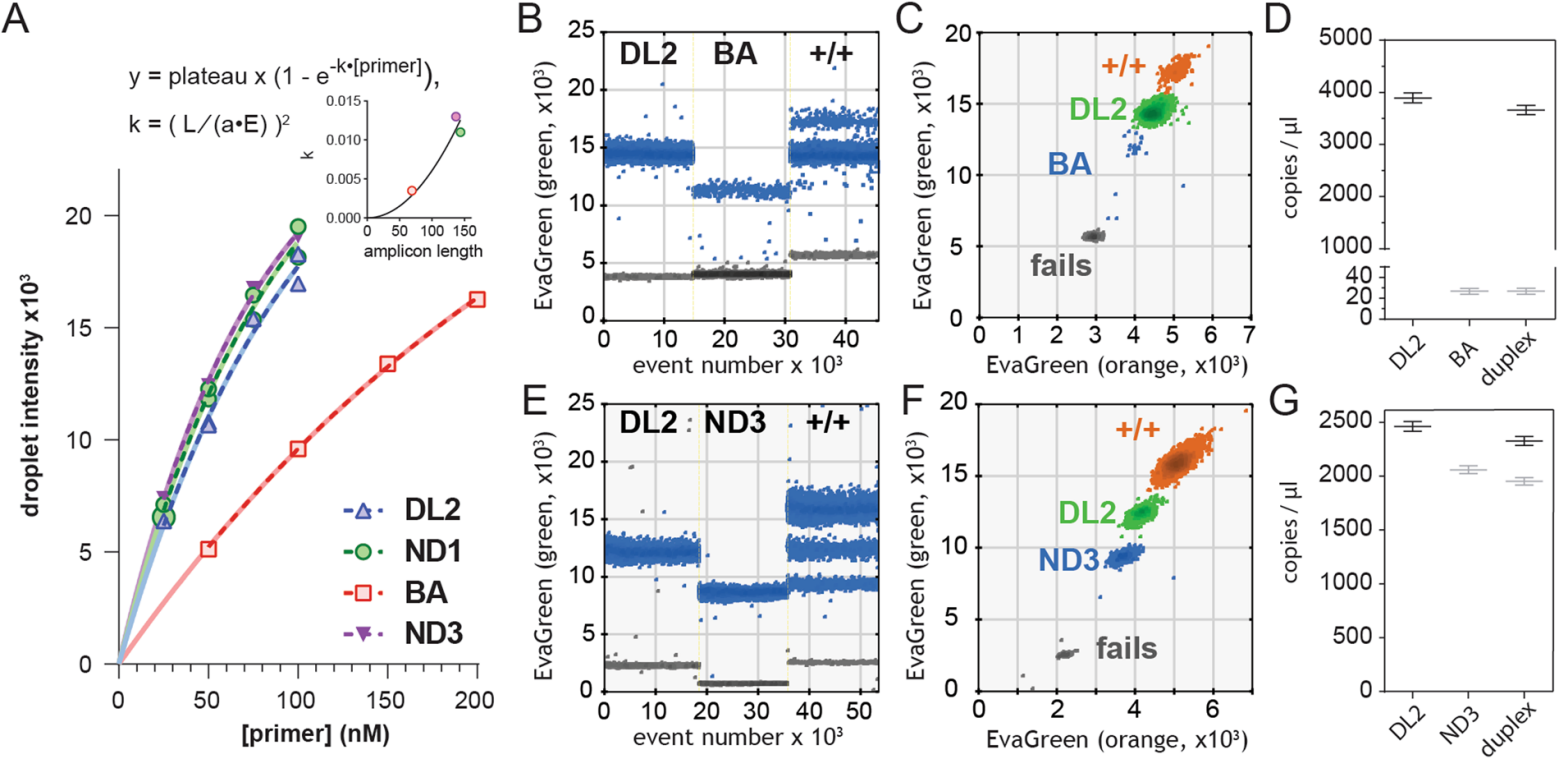
EvaGreen duplex reactions do not affect calculated template concentration. (**A**) Positive droplet intensity as a function of primer concentration and amplicon length. Amplicon lengths are 69 (BA), 137 (ND3), 144 (ND1) and 83 (DL2). Inset: equation 3, k fitted as a square law function of amplicon length (L). (**B**,**C**) Independent singleplex and multiplex reactions of DL2 and BA demonstrate 100 nM of each primer provides effective separation of droplet clusters in both 1D and 2D. E&F. Independent singleplex and multiplex reactions of DL2 (100 nM) and ND3 (40 nM). (**D**,**G**) Copy number for singleplex (n = 3) and multiplex (n = 3) reactions for both assays. (**B**,**C**,**E**,**F**) EvaGreen intensity collected on QX200 filters suited for FAM (green) and HEX (orange) dyes.

The EvaGreen multiplex assay requires discrimination of three to four droplet populations: fails (no template), droplets containing either of two targets, and potentially a band of double-positive droplets. Distinguishing these populations is possible with EvaGreen because droplet intensity is determined by amplicon length, primer concentration and primer amplification efficiency^13^. Measured template concentration is unaffected by primer concentration in ddPCR^20^ (Fig. S2). The critical factor for a precise EvaGreen duplex assay was choosing primer concentrations that produced separated single-positive droplet populations and allowed for the discrimination of the two (or three) populations of positive droplets. Example for DL2/BA and DL2/ND3 are shown in Fig. 2. When the separation of single-positive droplet intensities was too large, the brighter single-positive droplets tend to overlap with double positives droplets (not shown). To streamline selection of primer concentrations, we plotted mean droplet intensity as a function of primer concentration in singleplex assay. These plots closely fit an exponential function of the form:

1 $$droplet\,intensity=plateau\,x\,(1-{e}^{-k\bullet [primer]})$$

We constrained the curves to pass through the origin and to have a plateau intensity of 30,000 fluorescent units and fitted the rate constant (k) for each pair of primers. Assuming that k is affected by amplicon length (L), primer efficiency (E), dye brightness and detector sensitivity (*a*), we modelled k as per Equation 2:

2 $$k={(\frac{L}{a\bullet E})}^{2}$$

By substitution, we derive equation 3:

3 $$droplet\,intensity=plateau\,x\,(1-{e}^{-{(\frac{L}{a\bullet E})}^{2}\bullet [primer]})$$

Measured template concentration is not affected by primer efficiency, but we modeled the relationship between primer concentration and droplet intensity as a diagnostic tool. Droplet intensity closely matched equation 1 (Fig. 2A, solid curves) and dashed lines show fits to equation 3, with L equal to the amplicon length for each primer and *a* empirically derived at 1235. This resulted in plateau and efficiency values for DL2 of 28284 and 0.70, BA of 32243 and 1.06, ND1 of 27742 and 0.96 and ND3 of 26395 and 0.98. Fits for equations 1 and 3 closely superimposed while providing reasonable values for plateau and efficiency. The four-point calibration curves allowed rapid selection of primer concentrations for future duplex assays.

Quantasoft analysis of duplex assays requires detection and analysis of EvaGreen intensity in both FAM and HEX channels. One-dimensional (FAM channel) views of duplex assays show that droplet intensity is consistent between singleplex and multiplex assays (Fig. 2B,E). Two-dimensional view facilitates gating droplet populations that are colour-coded as fails (grey), single positives (blue and green) or double positives (orange) (Fig. 2C,F). Template concentrations were consistent between singleplex and duplex assays (Fig. 2D,G). Similarly, for example, DL2:BA ratios were consistent between singleplex assays comparing the mean ratio of technical triplicates repeated in five experiments (133.6 ± 4.5) versus multiplex assays (technical triplicates) repeated in three experiments (135 ± 0.36). The multiplex reactions showed a 12-fold decrease in coefficient of variance, and the greatest source of error for these experiments was the addition of template to each reaction.

### Analyses of single versus multi-copy number genomic reference for mCN determination

**Figure 3:**
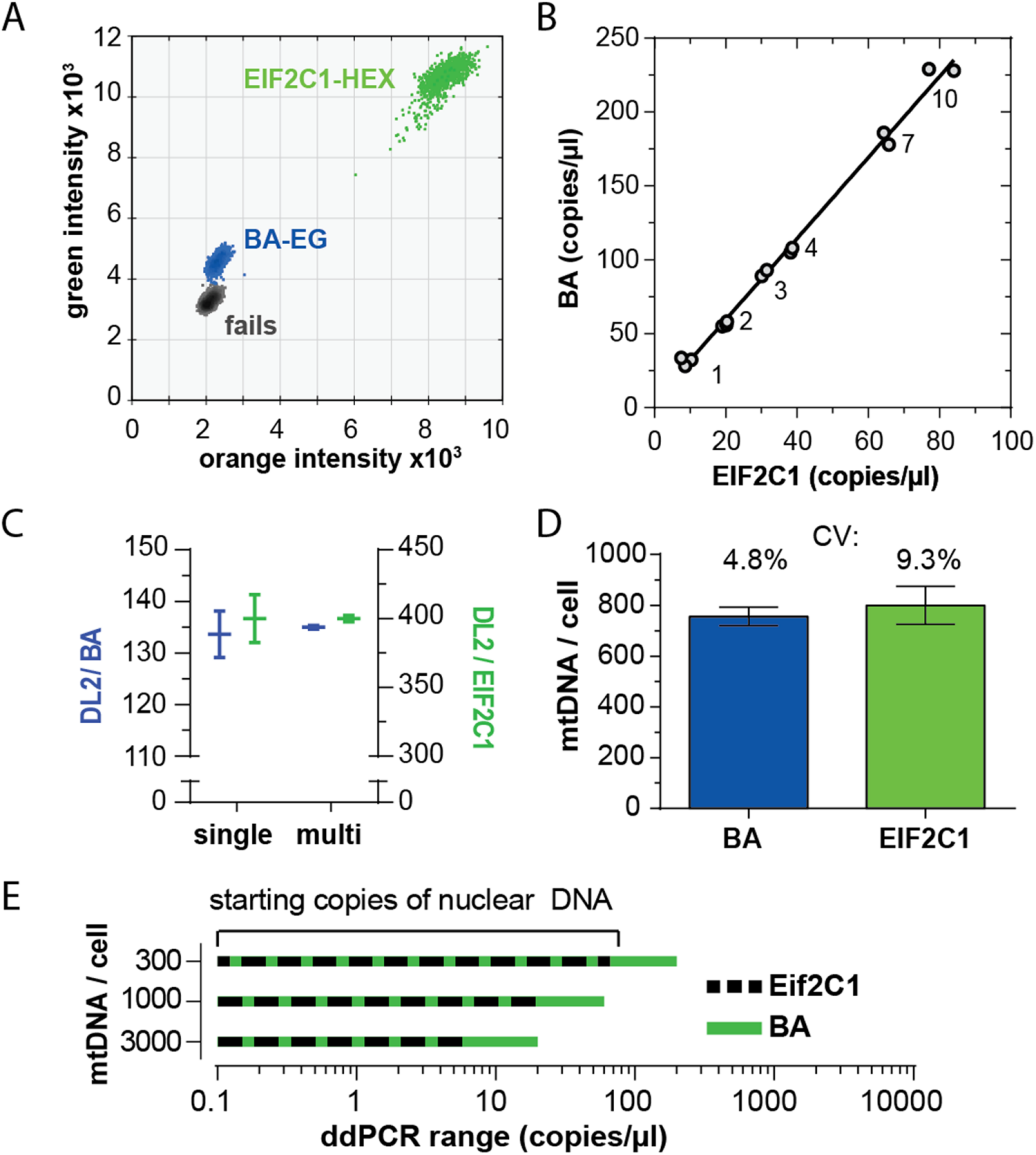
Copy number corrected ACTB provides increase precision and greater dynamic range of than a single copy number genomic locus. (**A**) Example droplet intensities of A7r5 gDNA amplified with ACTB (BA) using EvaGreen (BA-EG) and EIF2C1-HEX assay in the presence of EvaGreen. (**B**) BA:EIF2C1 ratio was calculated by linear regression from two independent experiments amplifying varying amounts of gDNA (experiment #1: 3 × 1.0, 2 × 3.0, 2 × 10.0 ng gDNA. Experiment #2: 2 × 2.0, 2 × 4.0 and 2 × 7.0 ng gDNA). Correlation coefficient (r) = 0.997, deviation of slope from zero, p < 0.05. (**C**) Comparison of BA and EIF2C1 as reference. Multiplex: BA (n = 14), EIF2C1 (n = 24). Singleplex: DL2 (n = 18), BA (n = 5), EIF2C1 (n = 21). (**D**) Calculation of mtDNA copy number using ACTB and EIF2C1 as reference genes adjusting for ACTB pseudogenes and 2 N ploidy via Multiplex Assay (n = 14 for ACTB, n = 24 for EIFf2C1). (**E**) Usable range of initial nuclear DNA concentration (copies/µl) in duplex ddPCR reactions for tissues with 300–3000 mtDNA copies/cell using EIF2C1 or BA as nuclear reference.

Measuring mCN typically relies on comparing mtDNA quantity to a nuclear reference gene. A PubMed search using the search terms “(mitochondria OR mitochondrial) AND copy number AND (D-Loop OR D Loop OR control region)” returned 82 titles. We retrieved 75 articles, and 63 used a specific nuclear gene to calculate mCN. The most frequently used genes were Actb (25 articles), beta globin (11 articles) and GAPDH (5 articles). Because ACTB and GAPDH have many pseudogenes that can confound absolute mCN determination^21^, many groups use single copy number genes (e.g. RPP30^22^, APP^23^ and beta globin^4^). However, the ratio of mtDNA to a single-copy nuclear locus can saturate ddPCR duplex assays in high mCN tissues^15^. We reasoned that using a nuclear reference with a known number of pseudogenes could extend the dynamic range of a ddPCR mCN assay. We found that absolute variance in measured template concentration (copies/µl) is similar at concentrations of 1–10 copies/µl (Fig. 3B). Therefore, using a pseudogene of known copy number should decrease the coefficient of variance of the denominator in equation 4 and increase mCN precision.

4 $$\begin{array}{cc}mCN\,per\,cell & \,=\,\frac{copies\,mitochondrial\,gene\,per\,\mu l\,reaction\bullet 2\,nuclear\,genomes\,per\,cell\bullet (1+no.of\,pseudogenes)}{copies\,genomic\,reference\,per\,\mu l\,reaction\,}\end{array}$$

EIF2C1 is a single copy number gene in the human genome, so we tested rat EIF2C1 as a potential single copy number assay using a commercially validated rat EIF2C1-HEX assay. A BLAST search for the region amplified by this assay (Rnor_5.0 5: 148233206–148233107, Rnor_6.0 5: 144465893–144465992) returned only the predicted region. We also screened the 5′ and 3′ 18–24 nucleotides of this region as primers using the *in silico PCR Tool (*http://rohsdb.cmb.usc.edu/GBshape/cgi-bin/hgPcr*)*, which predicted amplification of only the expected amplicon. Thus, the EIF2C1 assay likely provided a single copy number assay. We measured EIF2C1 and BA in multiplex reactions using EvaGreen supermix and observed a single cluster of fails, a single cluster of BA-EvaGreen positive droplets and a single cluster of EIF2C1-HEX positive droplets (Fig. 3A). We observed a linear relationship between EIF2C1 and BA copy number (slope = 2.8 ± 0.1, r^2^ = 0.99) and a mean EIF2C1:BA ratio of 3.0 ± 0.12 (mean ± SEM, n = 14.) (Fig. 3B). Rat liver genomic DNA (male and female, not shown) also had a mean EIF2C1:BA ratio of 3.0 ± 0.1. Thus, the BA primers amplified three targets per genome in a sex-independent manner.

We compared mCN in A7r5 cells relative to BA and EIF2C1 using singleplex and multiplex assays. We analyzed gDNA from a single A7r5 passage to minimize inter-sample variance. The coefficients of variance were smaller for DL2:BA and DL2:EIF2C1 ratios for multiplex versus singleplex assays (Fig. 3C), and the ratio of mitochondrial copy number to nuclear reference differed by a factor of ~3 for mtDNA/BA (135.0 ± 0.4, n = 4) versus mtDNA/EIF2C1 (400.0 ± 2.4, n = 3). Using equation 4, this was equivalent to 750–800 copies/cell. Using a nuclear reference with three copies versus a single copy number reduced the mCN coefficient of variance from 9% to 5% (Fig. 3D). Figure 3E further illustrates usable starting quantities of gDNA for BA versus Eif2C1 mCN assays in tissues with a range of mCN (see discussion).

### Estimating single cell mCN

**Figure 4:**
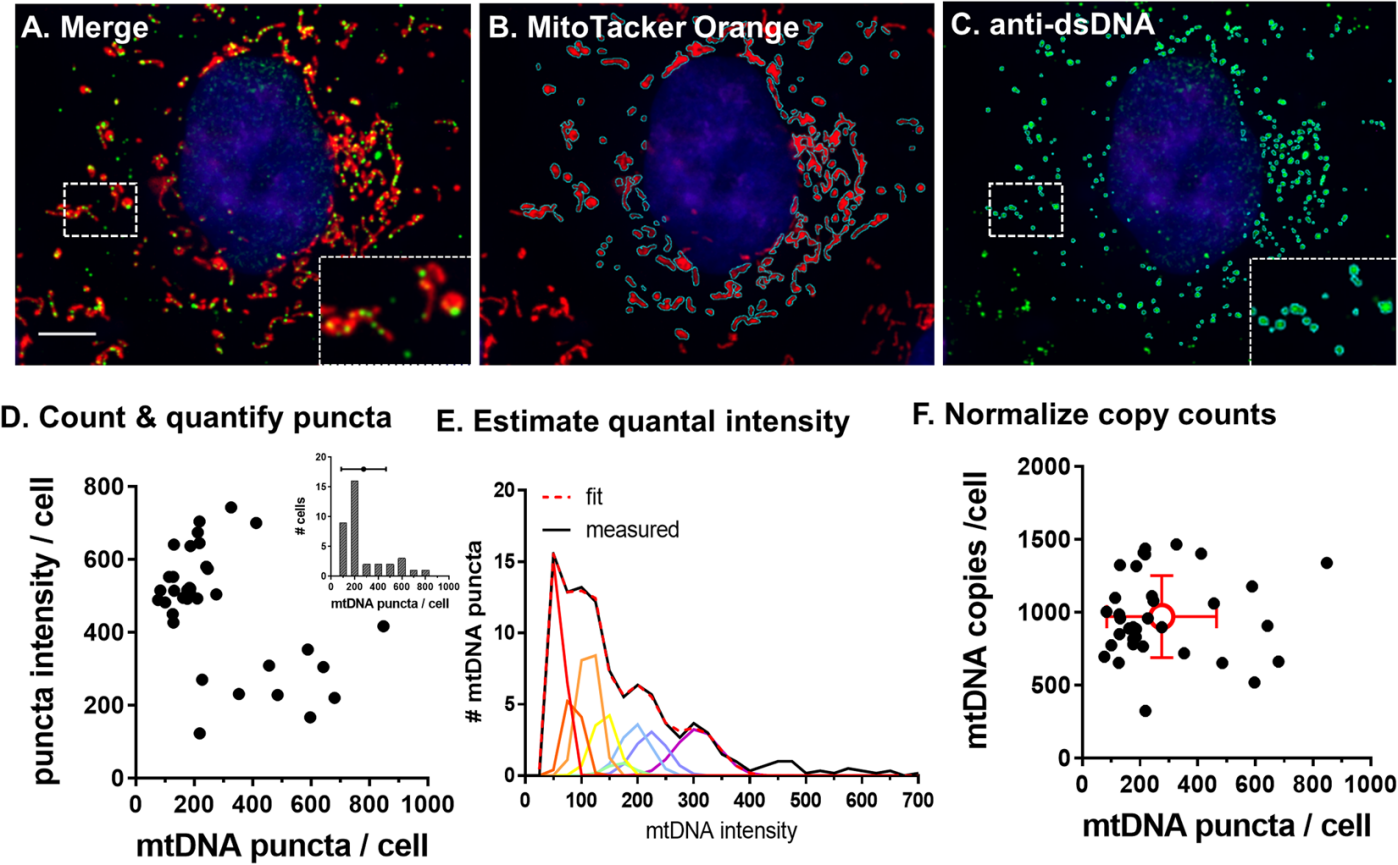
mtDNA copy number estimated by counting dsDNA puncta in mitochondrial corroborates absolute CN quantification by ddPCR. A-C. A7r5 cells labelled with (**B**) MitoTracker Orange, fixed and stained with (**C**) anti-double-stranded DNA antibody to detect mtDNA were imaged with a 100 × 1.45NA objective. dsDNA puncta were analyzed with ImageJ using a custom written multiple-thresholds segmentation algorithm with cell ROIs drawn by hand to exclude the nucleus. (**D**) Puncta/cell as a function of mean puncta intensity per cell. Inset: Histogram of mtDNA puncta / cell showing mean and SE (dot and horizontal bars). (**E**) The intensity distribution of mtDNA puncta was fitted with a 10-Gaussian model to estimate single genome intensity. mtDNA copy number per cell is the sum of puncta intensities divided by the single genome intensity. (**F**) mCN calculated from the estimate of single nucleoid intensity reduced variance in the calculation of mtDNA copy number compared to counting of mtDNA puncta.

To validate mCN measured by ddPCR by an alternate method, we visualized mtDNA nucleoids by immunocytochemistry. We acquired epifluorescence Z-stacks (0.9 µm steps) of A7r5 cells immunolabelled for double-stranded DNA (dsDNA) and stained with MitoTracker Orange and Hoechst 33342. Stacks were reduced to single in-focus images using Extended Depth of Field processing (Fig. 4A)^24^ then rolling ball subtracted to reduce nuclear dsDNA fluorescence. The perimeters of cells were estimated by the MitoTracker labelled area, and the cytosolic regions of interest (ROI) were drawn by hand in ImageJ to exclude the nucleus. Within each cellular ROI, sub-regions of interest surrounding mitochondria and puncta of dsDNA were generated semi-automatically using a multiple-thresholds macro (https://imagej.nih.gov/ij/macros/Mulitple_Thresholds.txt) to rapidly identify circular puncta of dsDNA. The majority of dsDNA puncta were associated with MitoTracker labelled mitochondria (Fig. 4A inset). We measured 275 ± 190 (mean ± SD, n = 36 cells) dsDNA puncta per cell, with a skewed distribution (Fig. 4D inset), and a roughly reciprocal relationship between puncta number and mean puncta intensity. Extending the approach of Di Re *et al*.^23^, we assumed that puncta represent nucleoids containing 1–10 mitochondrial genomes^25^ and that the variance (sigma) in intensity will scale with genome copy number. We modelled the distribution of puncta intensity in each cell (Fig. 4D) as the sum of 10 Gaussian distributions (coloured curves) with means constrained to be integer multiples of a fitted quantal intensity and sigma values modelled as the unitary sigma multiplied by the square root of the n^th^ Gaussian. Quantal intensity and variance were held constant between cells that were fixed and imaged in a single batch. The fitted distribution (dashed red line), closely matched the observed distribution. We fitted curves using Prism GraphPad to minimize mean square error between the observed and fitted curves by adjusting the unitary intensity, sigma and number of puncta with 1–10 mtDNA copies. We calculated mCN as the sum of puncta intensities for a given cell divided by the quantal intensity. This analysis produced a normal distribution of mCN averaging 970 ± 280 (mean ± SD, n = 36 cells) mtDNA copies / cell (Fig. 4F), in close agreement with mCN estimates using the ddPCR EIF2C1 assay and providing an estimate of variance in mCN between individual cells.

### Comparison of the D-Loop, non-coding region and ND1 as mitochondrial reference loci

**Figure 5:**
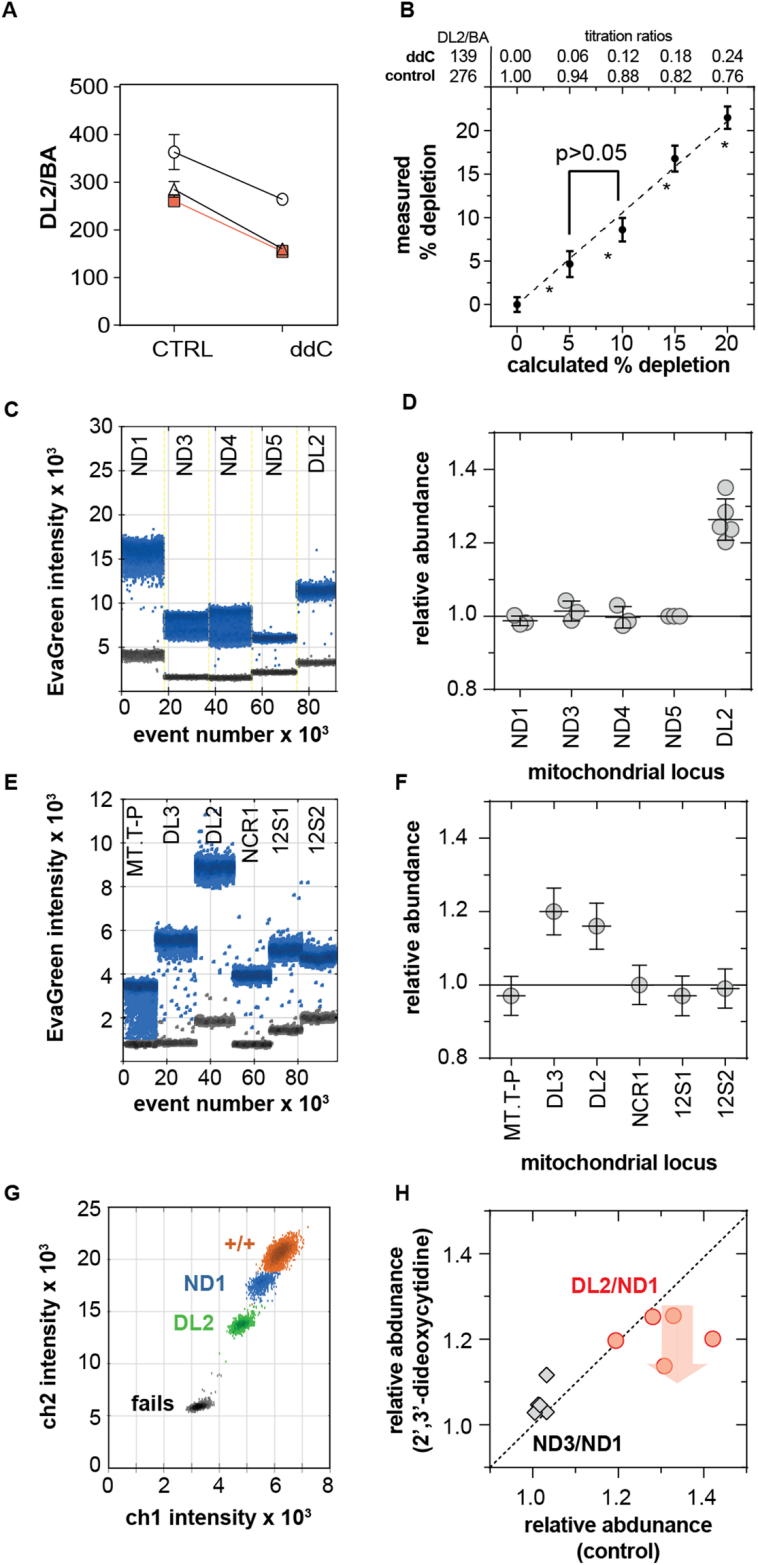
Assay sensitivity and anomalous DL copy number. (**A**) DL2:BA ratio from multiplex assay of gDNA from A7r5 cells grown in normal culture media without (CTRL) or with 2,3-dideoxycitidine (ddC, 30 µM) for 2–14 days. Error bars are standard error. (**B**) Assay sensitivity was assessed by titrating gDNA from control and ddC-treated cells to create known levels of mtDNA depletion. *All pair-wise comparisons are significantly different other than for the 5% and 10% groups. C. Singleplex droplet distributions for ND and DL2 primers amplifying A7r5 gDNA. (**D**) Copy numbers from C normalized to ND5 for each experiment. (**E**) Representative singleplex droplet distributions for DL2 and flanking primers (see Fig. 1) using A7r5 gDNA. (**F**) Copy numbers from E normalized to NCR1. Error bars are 95% confidence interval of Poisson-corrected concentrations (**G**) Typical droplet intensity distribution for the ND1-DL2 assay. (**H**) Sister cultures of A7r5 cells treated with ddC (as in A) or vehicle (control), and analyzed for DL2:ND1 and ND3:ND1 ratios. Dotted line indicates no difference. Data points below line show depletion by ddC.

Literature analysis revealed that the ND1 gene and the D-Loop portion of the non-coding region are commonly used as reference loci for mCN calculation. The DL2 and ND1 primers produced a single distribution of positive droplets, indicating a single length of amplicon for each (Figs 2B,E, 5C). To assess the sensitivity of the EvaGreen duplex assay, we titrated A7r5 gDNA from naïve cells and cells treated with 2,3,-dideoxycitidine (ddC, 30 µM) for 2–14 days to impair mtDNA synthesis^26^. ddC decreased the DL2:BA ratio by 37 ± 8% (n = 3 independent replicates, 4 technical replicates) (Fig. 5A). Naïve and ddC-treated gDNA were titrated to simulate the depletion of mCN at 5% intervals, measured as the DL2:BA ratio in technical quadruplicates (n = 4) (Fig. 5B). A 5% change in mCN was statistically detectable by One Way ANOVA (Scheffe *post hoc* test, SPSS Statistics 20, IBM) in all but the 5–10% titrations. The coefficients of variation for the 5% and 10% titrated depletions were larger than for undiluted DNA, suggesting that pipetting error significantly contributed to total variance. A 5% limit of detection is a conservative estimate of assay sensitivity.

We measured ND3:DL2 and ND4:DL2 to determine if the ddPCR assay could detect the minor fraction of mtDNA that carry the common deletion. The DL2:ND3 (Fig. 2G) and DL2:ND4 (not shown) ratios ranged between 1.1–1.4, similar to Philips *et al*.^8^. These ratios were not due to high common deletion heteroplasmy. Endpoint PCR using primers flanking the common deletion region produced a major band at the expected ~5600 bp length and faint bands at 500–750 bp consistent with common deletion (Fig. S3). The total fluorescence of the common deletion bands versus the full-length amplicon, adjusted for amplicon length suggested a heteroplasmy of 0.6% in A7r5 cells and 0.7% in aorta. qPCR reactions with DL2 and ND3 primers also indicated a deletion frequency in A7r5 cells of 0.5–0.8% (min-max, mean of triplicate reactions in 3 independent experiments), consistent with values reported for rat liver^19^. Using similar deletion-flanking primers for ddPCR results in extensive rain (incomplete elongation) and heteroplasmy values beyond 100%, so we did not further optimize ddPCR for common deletion analysis. However, comparing singleplex ddPCR reactions for DL2, ND1, ND3, ND4 and ND5 showed that the DL2 abundance was anomalously high (1.26 ± 0.05, n = 5) relative to ND1 (0.98 ± 0.01), ND3 (1.01 ± 0.03), ND4 (1.00 ± 0.03) normalized to ND5 (n = 3 for each) (Fig. 5C,D).

We compared the DL2 copy number in A7r5 and rat liver gDNA to a series of sites within and flanking the non-coding region as illustrated in Fig. 1 (primer sequences in Table 1). Normalized to NCR1 primers, the DL2 and neighbouring DL3 primers had mean copy numbers of 1.18 (Fig. 5E,F) and 1.45 (not shown) in A7r5 and liver gDNA. Primer sites immediately 5′ of DL3 and 3′ of DL2 failed to prime. Further 5′ and 3′ of DL3 and DL2 were large stretches of low complexity sequence (labelled as “ncp” in Fig. 1) in which PrimerBlast could not define suitable primers for our reaction conditions. Copy number for primers spanning MT-T and MT-P tRNA genes (MT.T-P), the 3′ prime end of the non-coding region (NCR1) and two loci in the 12 S rRNA gene (12 S1, 12S2) amplified with copy numbers of 1.01 ± 0.05 (mean ± SD, n = 6) relative to NCR1. Compared to the previously published primer NCR-12S, DL2 had a copy number of 1.26 ± 0.05 in rat brain gDNA (not shown).

Grunewald *et al*. reported that primers targeting the D-Loop can amplify 7S DNA as a third strand of template^27^. Accordingly, D-Loop primers could report ~1.0–1.5-fold the abundance of other mtDNA loci depending on the fraction of mtDNA containing 7S DNA. If the high D-Loop copy number was due to 7 S DNA, then ddC should reduce the ratio of D-Loop copy number relative to the rest of the mitochondrial genome^26^. We compared ND3:ND1 versus DL2:ND1 ratios in ddC-treated versus naïve cells in four passages of A7r5 cells. Figure 5G shows gating of the DL2:ND1 assay. Plotting the ND3:ND1 ratio versus the DL2:ND1 from naïve versus ddC-treated sister cultures illustrates: (i) the variability of the DL2:ND1 ratio between passages of cells and (ii) the selective effect of ddC on the DL2:ND1 (Fig. 5H). In sister cultures, the ND3:ND1 ratio was 3.2 ± 3.0% higher in naïve versus ddC-treated cells, whereas the DL2:ND1 ratio was 7.2 ± 6.8% lower in ddC-treated cells (p = 0.007, n = 5, paired t-test).

### D-Loop copy number

**Figure 6:**
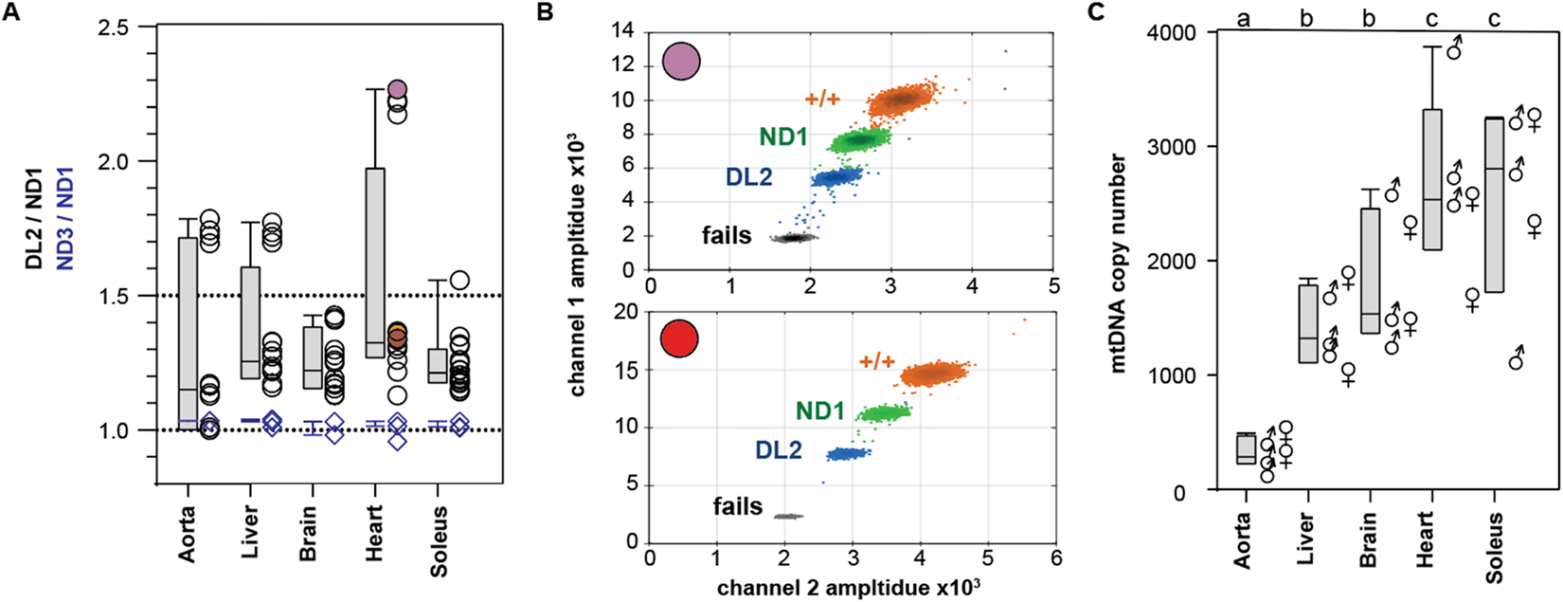
Variance of DL2:ND1 ratio and mtDNA copy number in different rat tissues. (**A**) DL2:ND1 (gray bars, black circles) and ND3:ND1 (blue bars, blue diamonds) ratios in gDNA isolated from rat tissues isolates from each of five rats. Box plots: line – median, box – interquartile range, whiskers – 10^th^ and 90^th^ percentiles. (**B**) 2D droplet intensity plots showing manual gating of droplets for samples with a high (top) and low (bottom) DL2/ND1 ratios. (**C**) mtDNA copy number measured as the ND1:BA ratio using equation 4, assuming two BA pseudogenes. Box plots as in A. Male (n = 3) and female (n = 2) symbols show mean of technical quadruplicates. Groups sharing the same letter (top) are not significantly different from each other by one-way ANOVA and Bonferroni corrected post-hoc t-test (group-wise α = 0.05).

We measured the anomalous D-Loop copy number in gDNA from aorta, brain, heart, liver and soleus skeletal muscle in triplicate or quadruplicate (Fig. 6A). The ND3:ND1 was similar between tissues (1.01 ± 0.02, mean ± SD, triplicates from five tissues). DL2:ND1 was greater than one in almost all cases. A two-way ANOVA (JMP 13.0) showed significant differences between individual rats (F(2,16) = 200, p < 0.001) and tissues (F(3,16) = 177, p < 0.001) and a significant interaction term (F(11,16) = 187, p < 0.001). The mean DL2:ND1 ratio between tissues ranged from 1.24 ± 0.10 (liver) to 1.52 ± 0.42 (heart) with coefficients of variation ranging from 8.5–27%. Between rats, mean DL2:ND1 ratio across tissues ranged between 1.17 ± 0.07 to 1.58 ± 0.45 with coefficients of variation ranging from 5.5–28% (not shown).

### mCN in rat tissues

Having established ND1 as the preferred mitochondria locus for mCN measurement, we characterized mCN in the same five tissues (Fig. 6C). Aorta has the lowest mCN (334 ± 131, mean ± SD, n = 5 rats in technical quadruplicates) followed by liver (1421 ± 354) and brain (1834 ± 585) then soleus (2547 ± 868) and heart (2673 ± 793). We did not detect an obvious effect of sex on mCN (two-way ANOVA: Tissue F(4,4) = 48.3, p < 0.001, Sex F(1,1) F 0.048, p = 0.49), but a marginal interaction was found for Tissue*Sex interaction for the female heart tissue (F(4,4) = 3.62, p = 0.009, Tissue[Heart]*sex[female] p = 0.0004)). This was based on only two female rats and may not be reproducible.

## Discussion

Droplet digital PCR assays have been described for mCN using hydrolysis probes, but the use of hydrolysis probes and limited dynamic ranges associated with using single copy number nuclear reference loci present potential limitations to their wide spread use^15,28^. We have characterized a single-dye absolute mCN assay using ddPCR, and illustrate how a multiple-copy number nuclear reference can extend the range of mCN that can be measured in a duplex ddPCR reaction. We report a complementary microscopic approach to estimate mCN that closely agrees with ddPCR. The absolute mCN that we measured in several rat tissues using ddPCR corroborated with a small number of other reports measuring absolute mCN in rat tissue^15,22,28^. More importantly, we demonstrate that the mitochondrial D-Loop, but not other regions of the non-coding region, is amplified with a highly variable anomalous ratio relative to other loci on mtDNA and thus should be avoided for analyses of mCN.

Assays for absolute mCN determination have been reported using qPCR, digital PCR and high-throughput sequencing^8,15,29,30^. Compared to qPCR, ddPCR natively reports absolute template concentration and is less expensive because serial sample dilutions are not required^11^. We achieved further cost-savings over a duplex probe assays by using an EvaGreen multiplex assay. The EvaGreen duplex assay costs ~CDN$11.00/sample (technical triplicates) versus ~$16/sample for a probe-based duplex ddPCR assay and $32/sample (technical triplicates x 4 dilutions) for a duplex qPCR assay. This represents a saving of CDN$672 for 32 samples with EvaGreen duplex versus qPCR. The mCN we observed for liver, skeletal muscle, brain and heart were similar to those reported using duplex probe-based assays and high-throughput methods^15,30,31^, with a similar limit of detection^8,32^.

Previous descriptions of multiplex EvaGreen assays leveraged amplicon length to distinguish the positive droplets from two targets^13^. We exploited the exponential relationship between primer concentration and droplet intensity as a basis for a duplex assay (Fig. 2)^20^. The square law relationship between the exponential constant k (Equation 1) and amplicon length might have been expected given the doubling of product with each PCR cycle. We assumed that k would also be affected by primer efficiency, such that low primer efficiency would increase k, providing a measure of primer quality. Analysis of droplet intensity at four or more primer concentrations permits close fitting of the primer concentration-intensity relationship such that optimal duplex droplet intensities can be determined from the individual calibration curves. We define optimal separation as the minimal separation of mean droplet intensities that provides minimal overlap of the two droplet populations. This ensures that double positive droplets do not overlap with the brighter populations of single-positive droplets. This optimization can be completed for 8–12 primers (2–3 replicates of each of 4 concentrations per primer) in a single 96-well plate, allowing subsequent mix-and-match creation of duplex assays.

Using a nuclear reference with a known number of pseudogenes allows a wider range of mCN to be measured in a single duplex ddPCR reaction (Fig. 3E). By corollary, ddPCR provides a simple method to determine pseudogene copy number if one has a single copy number assay for comparison. We observed a pseudogene number of two for the BA primers. Sun *et al*. identified 64 human and 69 mouse ß-actin pseudogenes using a BLAST search against mRNA sequences (i.e. exonic DNA)^21^. If rats have a similar number of *Actb* pseudogenes, one explanation for our primers not amplifying more pseudogenes is that our primer binding site is 111 bp 3-prime of the annotated A*ctb* gene (NC_005111.4, see Fig. 1). Alternatively, the EIF2C1 assay might amplify more than a single locus, causing us to underestimate the BA copy number and mCN. This is unlikely for the following reasons: 1) a Primer BLAST search of the rat genome returned two non-*Actb* hits using our BA primers, 2) ddPCR showed a relative copy number of 3:1 for the BA primers versus the EIF2C1, which in humans is a single copy number gene, 3) the mCN we calculated using EIF2C1 closely matched the mean copy number estimated from imaging mtDNA in intact cells, and 4) our estimates of mCN are in close agreement with those reported by others using ddPCR and high throughput sequencing^15^. Thus the most likely conclusion is that the EIF2C1 assay amplifies a single locus and that the BA primers amplified two pseudogenes.

The precision of ddPCR decreases rapidly when template concentration exceeds ~4000 copies/µl due to a steep inflection in the relationship between the portion of positive droplets and template concentration^16^. Thus, we would have arrived at similar mCN values if we used EIF2C1in place of BA, but with a higher variance in mCN. To illustrate, a discrepancy of 100 gated positive droplets out of 16000 droplets at 3000 copies/µl imposes a ~3% error in concentration. At 5000 copies/µl the same 100 droplet discrepancy causes an ~11% uncertainty. In contrast, at low concentrations of template (<60 copies/µl) the relationship between the fraction of positive droplets and copies/µl is linear. Therefore, in tissue like heart, with ~ 3000 copies/cell, one would need to dilute total DNA to achieve ~2 nuclear copies/µl of reaction if using a single copy number genomic reference. Assuming 16000 droplets are measured, 2 copies/µl equates to 30 positive droplets, where a gating discrepancy of 5 droplets imposes an 18% uncertainty in the mtDNA:nDNA ratio. In contrast, a genomic reference with three copies per genome (e.g. *Actb*) would have 6 copies/µl or ~80 positive droplets, such that a 5-droplet discrepancy imposes only a 6% uncertainty in mtDNA/nDNA ratio. Thus, using a multi-copy genomic reference proportionately increases the range of DNA that can be accurately assayed, which is of value in experimental designs in which a wide range of mCN is expected^15^.

Complementing ddPCR analyses of mCN, imaging mtDNA *in situ* provides a relative, single-cell measure of changes in mCN. Mitochondrial DNA has been imaged with hybridization probes^33,34^, DNA binding dyes^23,35^ and antibodies^25^. We found that immunocytochemistry provided consistent results, whereas the staining of DNA binding dyes (SYBR Green and PicoGreen) did not colocalize well with mitochondria in our hands (not shown). Individual nucleoids are ~100 nm in size, such that neighboring nucleoids appear as convolved puncta of varying size when imaged with diffraction-limited microscopy^36^. Past analyses of imaged mtDNA have measured puncta number and width^23,25^. By analyzing puncta intensity, which we assume to be proportional to the number of nucleoids in a cluster, our method produced a normal distribution of mCN between cells that permits the use of parametric statistics and that was not seen in the distribution of nucleoid number per cell (Fig. 4). The fact that optical estimation of mCN closely agreed with the independent measurement of mCN by ddPCR validates the accuracy of this approach. However, one limitation of this method is that the estimated single copy number intensity relies on the fitting a 13-parameter model that likely does not have a unique optimal fit. In future applications of this method, we would aim to calibrate optical analyses against mean mCN determined by ddPCR. This method provides a powerful approach for high content analyses by combining single-cell mCN analysis with additional optical assays of cell physiology.

Our initial interest in mCN was to study blood vessels because mitochondrial number and function decrease with age^3^, which presumably decreases mitochondrial mRNA levels^30^. Experimentally increasing mCN prevents vascular aging^37^, but normal vascular mCN is not well characterized. Our measures of heart mCN are consistent with human heart having ~2800 copies/cell based on high throughput sequencing^15^ and 6000 copies/cell by qPCR^30,31^. Our measures of skeletal muscle mCN were consistent with measures by qPCR, ddPCR and high throughput sequencing of ~3000 copies/cell^15,30,31^, though skeletal muscle mCN has been reported to be as low as 40 copies/cell^38^. Our measure of brain mCN (~1800) is also consistent with past estimates (800–2500 by qPCR, ~1100 by ddPCR)^22,30^. We observed considerable variance in mCN between tissues and individuals. With respect to aorta, mCN was two to three times higher in cultured A7r5 cells than aorta. While, the aorta contains several cell types, the majority of cells are smooth muscle, making this a reasonable comparison. In freshly isolated smooth muscle cells, mitochondria are sparse, punctate and largely immobile, and occupy ~10% of the cross sectional area of the cytosol^17,39,40^. In cultured smooth muscle, mitochondrial morphology changes to a dense, mobile network, occupying 20–30% of the cytosolic cross-sectional area^17,41^. Thus the three-fold increase in mCN between intact aorta and cultured A7r5 cells is consistent with previous reports of the extent of increase in mitochondria mass upon culturing smooth muscle cells.

D-Loop copy number was anomalous and experimentally labile relative to other loci on the mitochondrial genome. Several explanations could account for the non-unitary copy number. First, NUMTs could be amplified by the D-Loop (DL2 or DL3) primers. We identified NUMTs with close homology to the NCR-12S and DL2 amplicons. However, NUMTS should add a fixed number of “mitochondrial” copies per cell and could not account for variance in the DL:BA ratio between tissue, individuals and ddC treatment. Second, some mitochondria could harbor duplications of the D-Loop and non-coding region as seen in crane, lizard and deep-sea scallop;^42–44^ however, duplications are not present in three rat mitochondrial reference genomes (JX105355, NC_001665 and X14848). Third, the high and variable D-Loop copy number could be due to the amplification of 7S DNA. The fraction of mtDNA containing 7S DNA ranges from 0–100%^45^. If 7S DNA amplifies simply as a third strand of template, this would be evident as 0–0.5 extra copies per mitochondrial genome, consistent with the typical DL2/ND values that we observed. In a minority of samples, the DL2/ND ratio exceeded 1.5. These high values were not an artifact of failed or flawed assays, as each assay was carefully gated by hand and showed four, well separated populations of droplets (Fig. 6B). Grünewald *et al*. reported a similar minority of DL:ND1 (or ND4) ratio being close to 2.0 in human *substantia nigra*^27^. While the mechanism by which amplification of 7S DNA could produce DL:mtDNA ratios above 1.5 remains to be determined, our findings support Grünewald’s conclusion that the DL region is better suited to measuring the replication state of mtDNA than mCN.

Understanding that D-Loop loci can amplify at 1.0 to 1.5 times the actual mCN has two important consequences. First, using the D-Loop to study mCN or deletion frequencies could produce erroneous conclusions. We initially mistook the anomalous DL:ND3 and DL:ND4 ratios as a sign of high common deletion heteroplasmy, as have others^8^. However, absolute quantification of copy number revealed that DL copy number is high, rather than ND3 or ND4 being unusually low. Second, variability in the DL copy number related to cell cycle regulation and altered mtDNA replication, rather than a change in mCN, could account for the 10–20% change in “mCN” reported in several forms of cancer^46–48^. In this context, the DL:ND1 duplex assay we describe could be used in parallel with the ND1:BA primers to study mtDNA replication status, similar to the triplex qPCR assay described by Grünewald *et al*.^27^.

In summary, the ability to use a single DNA-binding dye to perform multiplex reactions makes ddPCR highly cost effective compared to probe-based duplex reactions, while offering precise and accurate mCN values. Based on our current results, we strongly discourage the use of the D-Loop in future studies of mCN. The assays described here can be readily adapted to other species or other preferred mitochondrial or nuclear loci, and the absolute quantification afforded by the mCN assay provides a simple method to calibrate optical measures of mCN in single cells.

## Electronic supplementary material

Supplementary Figure
