# Supplementary material for: Droplet digital PCR shows the D-Loop to be an error prone locus for mitochondrial DNA copy number determination
Source: Sci Rep. 2018 Jul 30;8:11392. doi: 10.1038/s41598-018-29621-1 (PMC6065360; doi:10.1038/s41598-018-29621-1)
Supplement: Supplementary file 1 — Supplementary Figure [file 41598_2018_29621_MOESM1_ESM.pdf]

Droplet digital PCR shows the D-Loop to be an error prone locus for mitochondrial DNA copy number determination:

Brian Li, Sonal Kaushik, Pola Kalinowski, BaRun Kim, Cynthia Gershome, K. Y. Joyce Ching, Damon Poburko

Supplemental Figures:

Figure S1

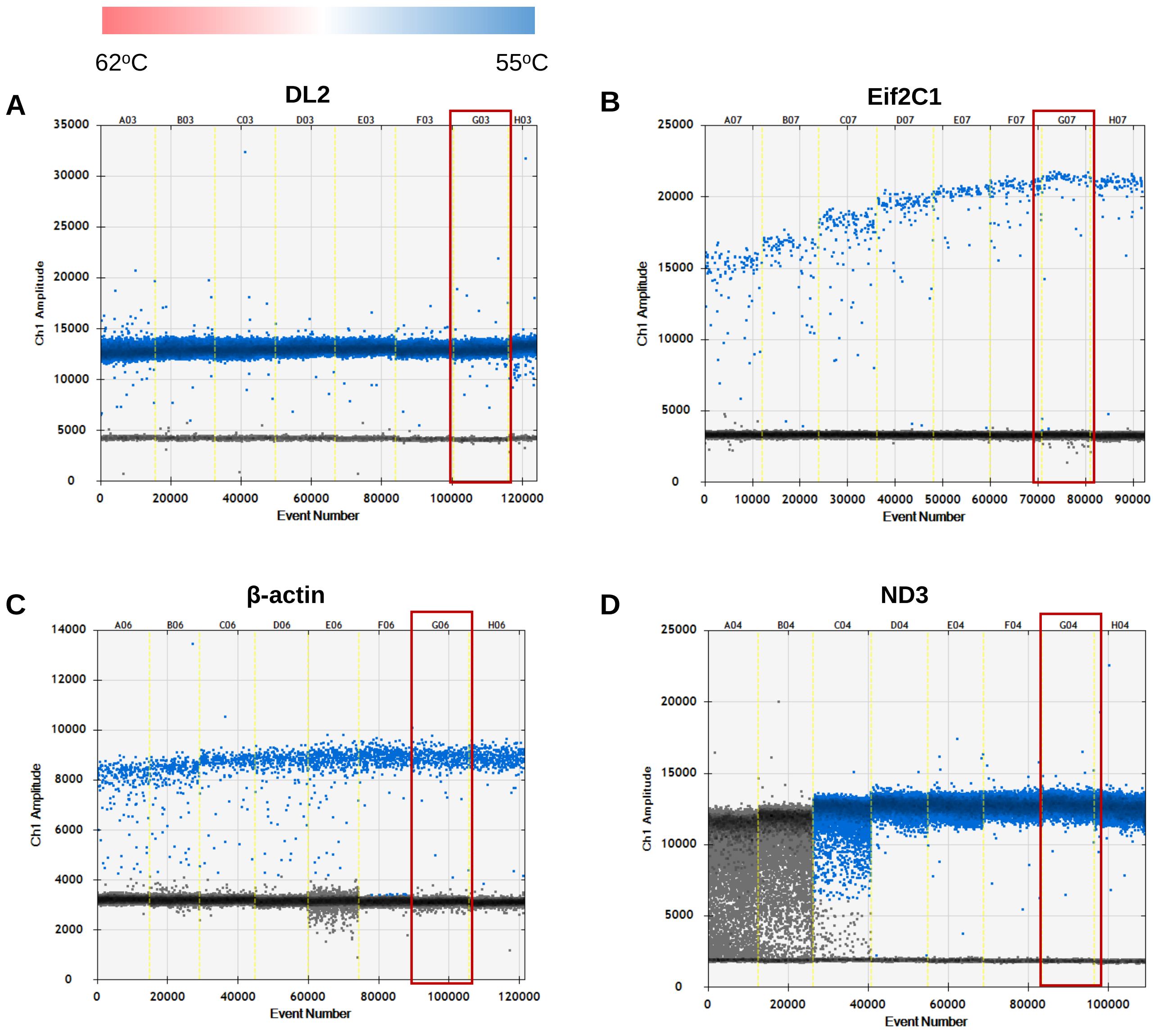

Figure S1. Optimization of annealing temperature for multiplex reactions.

Eight reactions for each primer and probe were run with a temperature gradient ranging from 55°C to 62°C in a 96 well plate. Standard ddPCR protocol was followed. From the raw data output above, optimal temperature was experimentally determined to be 56°C based on wells with the least rain between positive and negative droplets, an indication of effective annealing and extension of target DNA sequence. Rain is an indicator of incomplete amplification of the target of interest.

Figure S2

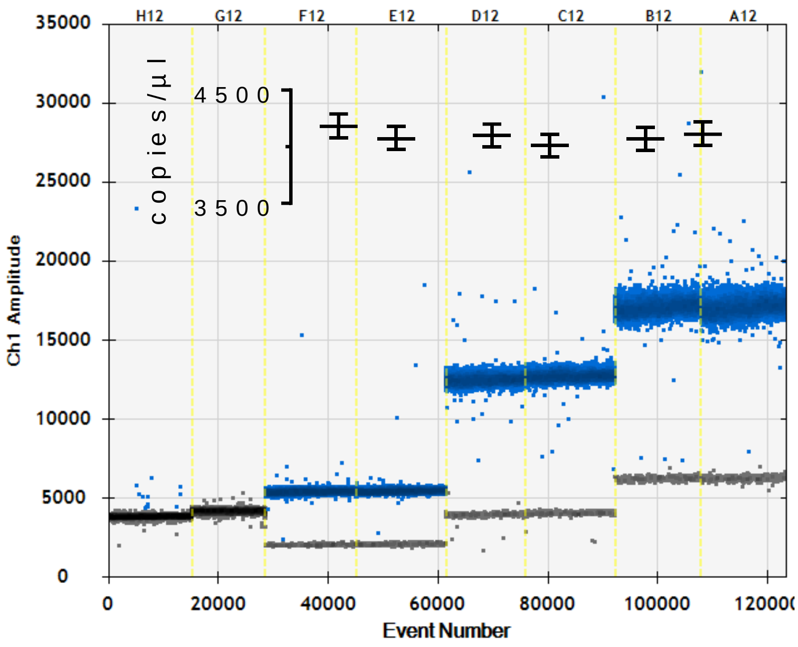

Figure S2. Primer concentration does not affect calculated template concentration.

A. Eight technical replicates containing 1.0 ng of A7r5 genomic DNA were amplified with varying concentrations of the DL2 primer (50 ,100 or 200 nM) or 50 nM without template (NTC: No Template Control). A plot of calculated copy number shows that copy number calculation is insensitive to primer concentration. Note that the Y-axis on the overlaid plot is highly truncated.

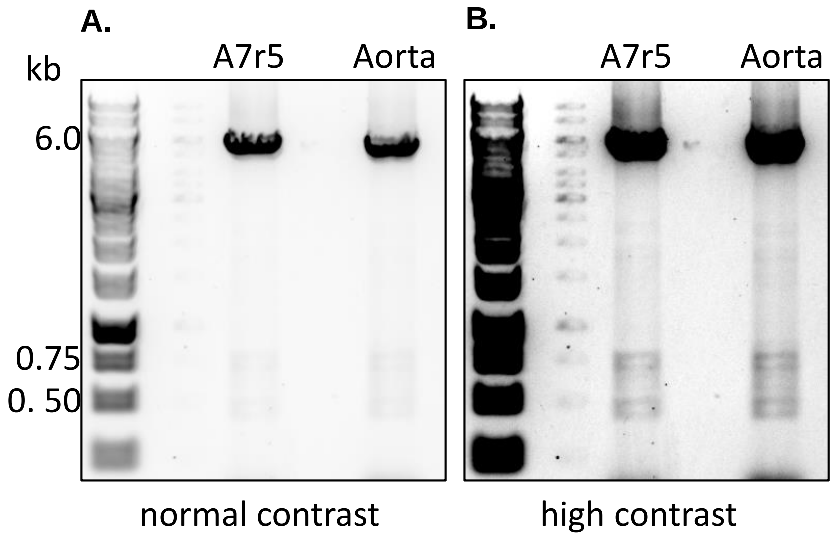

Figure S3. Mitochondrial Common deletion is detected by end-point PCR in A7r5 and aorta DNA.

Total Genomic DNA was isolated from A7r5 cells and aorta using the GeneJet Total Genomic DNA kit (Thermo). Primers flanking the common deletion region were (f) 5'-AAATTTCTTCCCAAACCTTTCCTG-3' and (r) 5'-TTTAGATAGTTGGGTTTGGGTGA-3'. 5 µl of each PCR reaction was run on a 1% TAE gel at 5.3 V/cm for 50 min. 5 µl of O'Gene 1kb Plus DNA ladder was run as a size standard. (A & B) – Images of gel at two contrast levels to visual ladder and ~6 kb bands without saturation (A) and enhanced contrast (B) to visualize minor bands representing amplification products of mitochondrial DNA having undergone the common deletion. Notably, two recombination sites appear to be detected.
